# Paliperidone palmitate-induced enuresis: a case report

**DOI:** 10.1192/j.eurpsy.2022.2063

**Published:** 2022-09-01

**Authors:** S. Cruz, N. Cunha E Costa, A. Morais, M. Mendonça, R. Trindade, S. Xavier

**Affiliations:** Hospital Garcia de Orta, Almada, Psychiatry, Almada, Portugal

**Keywords:** paliperidone, schizophrénia, enuresis

## Abstract

**Introduction:**

Schizophrenia is a severe mental illness that requires long-term treatment with antipsychotics and the intramuscular (IM) long-acting injectable (LAI) formulations may enhance treatment adherence. Some antipsychotics have been associated with enuresis, including atypical antipsychotics such as risperidone(6.2%), quetiapine(6.7%), olanzapine (9.6% ) and clozapine (20.7%) [1]. Although oral paliperidone has been related to urinary incontinence, there is only 1 report of urinary incontinence linked to monthly paliperidone palmitate [2]. [1] Harrison-Woolrych, M., Skegg, K., Ashton, J., Herbison, P., Skegg, D.C., 2011. Nocturnal enuresis in patients taking clozapine, risperidone, olanzapine and quetiapine: comparative cohort study. British Journal of Psychiatry 199, 140–144. doi:10.1192/bjp.bp.110.087478 [2] Karslıoǧlu, E.H., Özalp, E., Çayköylü, A., 2016. Paliperidone Palmitate-induced Urinary Incontinence: A Case Report. Clinical Psychopharmacology and Neuroscience 14, 96–100. doi:10.9758/cpn.2016.14.1.96

**Objectives:**

To establish an association between paliperidone palmitate and enuresis.

**Methods:**

Case report and a narrative review of the literature.

**Results:**

The patient was a 25-year-old healthy man when he was diagnosed with schizophrenia. Doctors prescribed paliperidone palmitate (LAI) 200mg monthly and he started to complain of enuresis. He was clearly suffering with this unpleasant and embarrassing adverse effect so the LAI was reduced to 150mg. Enuresis remained, so it was prescribed oxybutynin 20 mg/day and the patient improved.

**Conclusions:**

We reported a case in which enuresis is likely to be associated with high-dose paliperidone LAI (with no clinical evidence of an organic cause). To treat it, the most effective strategy was oxybutynin 20 mg/day. This case is also important to show the impact of this symptom, which is not actively investigated.

**Disclosure:**

No significant relationships.

